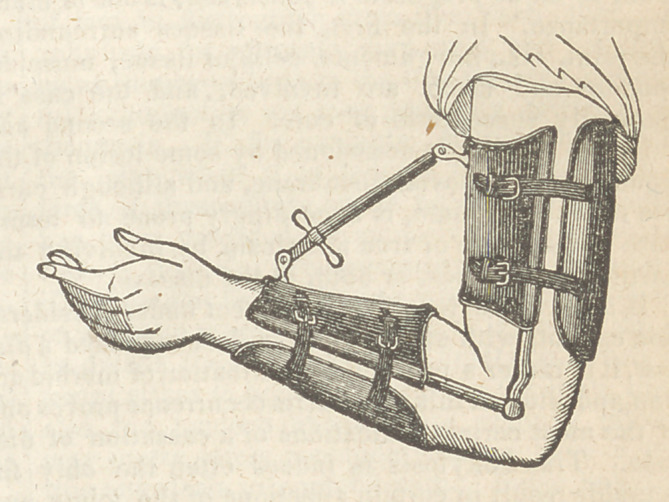# Jefferson Medical College

**Published:** 1843-03-18

**Authors:** H. T. Child


### CLINICAL LECTURES AND REPORTS.

### JEFFERSON MEDICAL COLLEGE.

#### CLINIC OF PROFESSOR MÜTTER.

*Dispensary of Jefferson Medical College, Jan.* 18, 1843.

(Reported by H. T. Child.)

#### LECTURE III.

CASE.—*False ankylosis of the elbow joint succeeding fracture of the condyles of the humerus, of three months standing, and materially impairing the usefulness of the whole limb—Treated with the screw of Stromeyer.*—The patient, a boy æt. ten years, received a fracture of the condyles of the left arm about four months since and was subjected at that time to the usual treatment for this injury, by the gentleman under whose charge he was placed. The bones united with some deformity, though not a great deal, and the integrity of the joint itself was well preserved. The natural motions in every direction, except *extension*, could be exerted but it was impossible to straighten the limb;—the forearm was flexed at nearly a right angle, and when an attempt was made to diminish the angle, the contraction of the biceps and brachialis internus muscles resisted the effort so completely that scarcely any alteration in the shape of the limb could be effected. There was no pain in the joint even when it was forcibly twisted and the bones pressed against each other, nor was there any swelling or deposite of callus to interfere with the motions, or contraindicate an immediate treatment of the case. The resistance to be overcome was seated in the tendons of the biceps and brachialis internus muscles, which were thrown into bold relief and became rigid whenever an attempt was made to straighten the limb.

This case, Professor M. remarked, would be a very tempting one, for those who resort to the knife on all occasions and at all hazards—and the tendons of the muscles in fault would be at once divided. But he wished the class to recollect what he had so often impressed upon them, that an operation, it matters not how trifling its nature, should always be considered the last resource of the surgeon, and ought never to be performed until all other means calculated to accomplish our end had failed, or unless, from the nature of the case, we could say that nothing but the knife would benefit the patient. In the present instance the operation would be very simple, but it would be painful and might result in serious consequences, and from the short duration of the contraction the tendons were not too rigid to resist *mechanical* means alone. He should, therefore, employ the treatment which he had used for some years in the management of such cases, which consisted in the application of an instrument (a modification of the screw of Stromeyer,) so constructed as to keep up gradual extension of the limb. Should this fail, he might then divide the tendons by passing a small knife between them and the integuments and cutting from without towards the joint, removing the difficulty at once. The instrument was then applied, and the screw turned until the child complained of the extension. (*See fig.*) The screw is to be turned daily a thread or two, until the limb is straightened and then its action must be reversed, the arm being gradually brought back to its first position; after this the limb must be alternately extended and flexed several times a day. By doing this and at the same time making use of the warm bath, friction with oleaginous mixtures, and covering the parts with oiled silk, we secure free motion to the joint, and accomplish a perfect cure. Professor M. next proceeded to make some remarks on ankylosis.

The term ankylosis is derived from the Greek word *ανχυλος*, signifying *bent* or *crooked*, and is employed to designate that condition of a joint in which its motions both active and passive are either partially or entirely destroyed, accompanying which loss there is usually a change in the natural shape of the part. It must be borne in mind, however, that often in ankylosis there is little or no alteration of the shape of the joint involved; it is neither *bent* nor *crooked* as the etymology of the term would indicate. The beautiful specimens of ankylosed hip, elbow and knee joints, contained in my collection, and to which your attention has already been directed on another occasion, prove this fact. As the stiffness may involve one joint or several, may depend on different conditions of the constituents of the part, and may also be either partial or complete, surgeons have divided this disease into several kinds.

When the stiffness is confined to but one articulation, and is dependent, as it usually is, on the influence of some local cause, the ankylosis is termed *partial* or *local*. When all or nearly all the joints are involved, as in the cases of Baron Percy, Bernard Conner, and others reported in the different surgical works, and the defect is the result of an internal or constitutional cause, it is called *general* or *universal ankylosis*.

Another division of ankylosis is based upon the degree of motion preserved, and the character of the tissue in which the resistance to motion resides. Thus, when all motion is lost and the articular facets are united by bone, cartilage, or dense fibrous tissue, we have a case of *true* or *complete ankylosis*. Mayo has divided this form of the disease into the *osseous*, *cartilaginous*, and *mixed*, inasmuch as either *bone* or *cartilage*, or *both together*, may form the uniting medium.

When the stiffness is dependent upon some defect of the soft tissues of the joint, either *extra capsular* or *intra capsular*, or of the *capsule* itself, and the joint is susceptible of slight motion at the time our examination is made, or, if this be absent, capable of being rendered moveable by proper subsequent management, the ankylosis is *false* or *incomplete*. Again, these false ankyloses may be divided into the *extra capsular*, *intra capsular*, and *capsular*. and this division, so far as prognosis is concerned, is one of much importance. In the first, the tissues surrounding the joint, viz., integuments, cellular tissue, muscles, tendons and fascia are involved, and the case is generally susceptible of cure. In the second and third the rigidity is occasioned by some lesion of the ligaments or synovial membrane, and although curable if taken in time, is exceedingly prone to terminate in incurable or true ankylosis, by involving the cartilages or bones, or both, in the disease.

It must be obvious that the defect under consideration cannot, with strict propriety, be considered a *disease*, it is rather a product or termination of morbid action, and often on this account its occurrence proves one of the most certain indications of a cessation of disease. True ankylosis is indeed often the only favourable result in certain affections of the joints, and we consider the case as *cured* when by our remedies we are able to bring about its occurrence.

Before undertaking to relieve a stiff joint, therefore, we must carefully investigate its causes, and ascertain whether or not it is proper to attempt a cure of the deformity to which it often gives rise.

*Causes.*—The causes which operate in the production of ankylosis are numerous, and most of them occasion the complaint by keeping the parts involved motionless, or nearly so, for a length of time. There are some, however, that seem to exert their influence under all circumstances; for example, *old age*, *chronic rheumatism* and *chronic gout*. It is true the joints usually involved from the operation of any one of these causes are those possessing comparatively but little motion, as those of the spine, pelvis, and some of the ginglymoidal; and the individuals themselves are forced to lead very sedentary lives, which will of course favour the occurrence of stiffness.

*True ankylosis* is also occasionally developed in the effort which nature makes to protect herself from harm in certain curvatures of the spinal column. This beautiful provision is well shown in several specimens in my collection taken from individuals affected with this complaint. Ledges of bone are thrown out in the hollow of the different curves, so that the spinal column is supported and prevented from yielding so far as to endanger life.

*True ankylosis* is, however, most generally the result of some disease of the synovial membranes, cartilages, or bones of a joint, although it may result from long confinement to one position, as has been clearly shown by Malgaigne, Teissier, and others. It is commonly believed that the ankylosis resulting from long confinement of a joint to one position, as in the treatment of fractures of the extremities, belongs to the class of incomplete or false; and this to a certain extent is true, but we often have produced instead, complete and incurable stiffness. This fact adds another to the long list of objections to the use of an *immoveable* apparatus, in the treatment of fractures.

*False ankylosis*, especially the extra capsular form, is developed by causes somewhat similar, although they produce lesions by no means so grave as those which are present in the true. The most common of all these causes is *rest*. When a joint, even although perfectly healthy, is confined to one position for any length of time, we find its integuments and the cellular tissue beneath them, contract and become more or less rigid; its ligaments and tendons also stiffen, and the muscles to which they are attached shorten, where the limb is flexed, and lengthen, when it is extended, and often undergo changes in their tissue, by which they lose their physical characteristics, and are rendered to a certain extent useless, while the synovial secretion is arrested or diminished. A good illustration of the influence of rest upon the joints is afforded by the stiffened limbs of the *Fakirs* of India, who actuated by religious motives condemn themselves to the observance of one position during their whole lives. The same thing is often seen after the treatment of fractures, luxations, or sprains, where the limb has been maintained in one position for too long a period.

Wounds followed by sloughing of the skin, burns, scalds, &c., in short, any injury likely to be followed by extensive cicatrization, may lay the foundation of extra-capsular ankylosis. You all know the power with which one of these cicatrices forms, often destroying or displacing some of the most important parts of the body. Here is a hand, the fingers of which nearly touch the back of the forearm in consequence of a burn. This drawing you recollect as the one taken from a patient whose chin was drawn down to the sternum by the cicatrix of a burn, and held there for twenty-seven years. Such cases can only be relieved by very extensive plastic operations.

Extra-capsular ankylosis is also sometimes brought on by the developement of an ulcer, or an abscess, or a phlebitis, or an angioleucitis, the result of some trivial wound or blow received upon the part. The complaint being generally confined to one side of the limb, there is contraction in this direction, produced partly by the patient's flexing the limb to take off pressure from the inflamed surface, and partly by the swelling itself displacing the tendons and fascia in the neighbourhood. By and by lymph is deposited, and unless the disease is cured, there remains permanent contraction of the limb, with motion in but one direction. This form is readily distinguished from that dependent upon shortening of the muscles by the following experiment. If we take hold of the limb farthest removed from the body and steady the one to which it is attached, and then attempt to separate them, or bring the member to its proper shape, we find in this form of ankylosis that the soft parts, although rigid and unyielding, are yet

*smooth* and *even* upon the surface. Now when the tendons or muscles are chiefly in fault, as in the case before us, and the same experiment is tried, we find them standing out in bold relief, as hard and as rigid as pieces of wire, while the soft parts in the vicinity are comparatively loose and yielding.

Different affections of the tendons or muscles, in the vicinity of a joint, may also cause extra-capsular ankylosis. A man, for example, receives a slight wound of a tendon or muscle; irritation or even inflammation of the part sets in, and the joint contracts by a shortening of the muscle alone. Sometimes the same cause may excite adhesions between the tendon and its sheath, or may even cause a sloughing of the same part.

Sometimes this form of ankylosis is dependent on a constitutional cause, as rheumatism or gout: and the boy presented to you the other day with telapes equinus from rheumatism, both of whose ankles were immoveable, was an instance of this kind. Often, too, a loss of muscular power, either by sloughing, or wounds, or paralysis on one side of the joint, by destroying, as it does, the balance of power which naturally exists, will bring on this form of ankylosis. The sound muscles being no longer opposed by their antagonists, pull the limb into an unnatural position; and as there is no power to overcome this influence, they keep it contracted, and gradually accommodate themselves in length to the altered condition of the parts, so that when we attempt to straighten the member we find it impossible to do so, until, by stretching or division with the knife, the shortened muscles are made to yield.

In certain forms of club-foot, torticollis, contraction of the fingers and toes, &c., this kind of ankylosis is encountered. In cases of long standing we invariably find the muscles and tendons of the weak side stretched and frayed out, and so much weakened that months and even years may elapse before they regain their natural tone and vigor. This should always be explained to the patient, who will be obliged to make use of artificial support for some time after the member has been restored to its natural shape.

Extra-capsular ankylosis is also the result of contraction of fascia, as we see in certain forms of contracted elbow, knee, and hands. It may also proceed from the growth of tumors, and the deposite of bone around the joint.

*Capsular Ankylosis.*—This form is generally the result of a severe strain or twist of a joint, in consequence of which the capsular ligament is more or less injured—a luxation or fracture near the joint, gout, rheumatism, wounds, and even rest may occasion the same thing. It is extremely difficult to distinguish this variety of ankylosis from the *intra-capsular*; but usually in the latter the cause operating is more violent, the stiffness is accompanied with more pain, and it is more difficult to move the joint. When a capsular ankylosis is examined by dissection, we find the fibrous tissue of the capsule thickened and hardened, and sometimes converted here and there into cartilage or bone.

*Intra-Capsular Ankylosis* is the result of some disease of the synovial membrane, usually acute or chronic inflammation, or their results; but the peculiar degeneration of this tissue, so well described by Sir Benjamin Brodie, may also lay the foundation of the complaint. The bond of union here is nothing more than organized coagulable lymph, which sometimes stretches across the joint from bone to bone, in bands or cords; at others it is deposited in patches, and, in a few rare instances, has been found spread over nearly the whole of the articulating surfaces of the joint—thus glueing the bones, as it were, to each other.

The history of the case will generally enable us to decide as to the precise nature of the bond of union; but it should always be borne in mind that synovitis may lay the foundation of capsular, or even extra-capsular ankylosis, producing, as it often does, sub-inflammation of the cellular and fibrous tissues about the joint, and also the peculiar contraction of the muscles and tendons usually met with in diseases of the articulations. It also frequently gives rise to true ankylosis—the disease extending to the cartilage and bones. The extreme rigidity of the joint, the apparent soundness of the tissues around it, the swelling which, in the first stage of the disease, is usually present, the pain excited by moving the articulation, or pushing one bone against the other, the character of the cause, and the fact that such cases are usually preceded by all the symptoms of inflammation of the synovial membranes, all serve to indicate to us the variety of the affection.

*Liability.*—It is usually stated that the ginglimoid articulations are more liable than the orbicular to both true and false ankylosis; and the observation is correct, owing chiefly to the circumstance that the former are more exposed to accidents. Their large articulating surfaces, and the number of tendons and fascia by which they are surrounded, also predispose to the occurrence of the disease.

*Diagnosis.*—There is scarcely any difficulty in distinguishing the different forms of ankylosis from other complaints, but it often demands a great deal of tact to distinguish them from each other. False ankylosis, for example, may exist, and yet the joint be as immoveable as in the true variety; but by careful examination we may generally arrive at a just diagnosis. If, for example, there is no motion in the joint, when it is twisted or turned with great force, if these efforts excite no pain, if the stiffness has been preceded by extensive intra-capsular disease, if the joint is comparatively but little swollen, if the patient feels a jarring when the limb is struck, or when he takes a false step, if it be in the leg, if he dreads these shocks, and finally, if, when we attempt either to bend or straighten the limb, the muscles and tendons about it are scarcely moved, we may pretty safely conclude that we have a case of true ankylosis to contend with. False ankylosis is also sometimes confounded with a rigid condition of the muscles surrounding a joint, which suffers from some acute or chronic inflammatory disease. A case of this kind was recently shown me by my friend Dr. Rodman, and it occurred in the son of Mr. B——, of Schuylkill Eighth street. This lad had suffered from coxalgia for some months, and his limb was shortened seven inches—the thigh flexed at a right angle with the pelvis, and no perceptible motion in the hip-joint. At first I was under the impression that true ankylosis had taken place, but on a more careful examination it struck me that it might possibly be a case of simple false ankylosis from rigid muscles. I therefore applied an apparatus by which moderate and constant extension of the limb might be kept up, and had the satisfaction to find that in the course of a few days, without pain, fever, or any inconvenience, the limb was reduced to the plane of its fellow, and within one inch of its natural length. The details of this case will be given on another occasion. The fact that this condition of the muscles may give rise to complete immobility should be constantly borne in mind in our examination of stiffened joints.

In forming a diagnosis between the different kinds of false ankylosis, we must take into consideration the history of the case. If the rigidity is the result of *rest*, *cicatrices*, affections of the *subcutaneous cellular tissue*, *spasm* of the *muscles*, *slight wounds of the tendons*, or *injury of the fascia*, it is probably *extra-capsular*. If caused by a *sprain*, or *punctured wound*, or a *blow*, it is *capsular*; and finally, if *acute* or *chronic synovitis*, or *gout*, or *rheumatism*, or *slight disease* of the *cartilages* or *bones* has existed, we shall have the *intra-capsular* form.

*Prognosis.*—The prognosis in this affection varies with the character of the lesion, whether it be true or false; and if false, the nature of its cause, the duration of the case, the age and health of the patient, and the joint involved.

The prognosis, as regards a cure, is always most unfavourable in true ankylosis; and, until very recently—indeed, until the publication on this subject by my friend Dr. J. Rhea Barton, of Philadelphia,—all such cases were ranked among the incurable forms of the affection; and even now the propriety of resorting to the operation of Dr. Barton must be the result of mature deliberation. Many cases give rise to so little inconvenience as scarcely to warrant the hazard of the remedy; while others, as those which result from caries, or extensive disease of a joint, should not, as a general rule, be touched, inasmuch as ankylosis is the most favourable termination of the disease; besides which, the operation may excite anew a disease in the part sufficiently intense to destroy the patient. When the stiffness is partial, the prognosis is usually more favourable, but even here we sometimes find it impossible to effect a cure. When it is dependent upon extra-capsular lesion, of no very long standing, and there is no loss of tendon or muscle by sloughing, the prognosis is favourable; but if the ankylosis be capsular or intra-capsular, unless the primary disease has been slight, or confined to the ligamentous tissue, as in gout or rheumatism, and the case of recent occurrence, we shall generally have great difficulty in accomplishing our object.

When the stiffness depends on the location of a tumour, &c. in the vicinity of a joint, the prognosis is generally favourable, inasmuch as we can remove the cause. The younger the person, the greater the probability of a cure in almost all cases; for, as we increase in years, the tissues become more rigid and unyielding. The general health, too, of the patient must be taken into consideration; there are some persons so irritable and prone to inflammation, that the slightest effort towards a cure, made either with the knife or mechanical means alone, is sure to excite disease. We should, therefore, carefully examine the case, and determine, as nearly as possible, the propriety of instituting any treatment before our attempts are commenced. In illustration of the importance of this cure, I may mention that several persons have either lost their lives, or been reduced to a very critical condition, by the attempts to cure false ankylosis by the screw. Prof. Pancoast mentioned to me a few days since, that he had been told of three individuals who had thus been destroyed. In my own practice I have been obliged, in two cases, to suspend the treatment, until the general health was so much improved as to justify the attempt to straighten the limb to be renewed. The prognosis is also modified by the joint involved; for we find it much more easy, as a general rule, to cure ankylosis of the *orbicular*, than of the *ginglymoidal* articulations. The function of the joint is also found to modify the prospects of cure; thus, where the articulations of the maxillary bones are involved, as in the cases of Cruveilhier and others, it will be found impossible, by any operation, to afford relief. In one case mentioned by Blackburn, the patient died from inanition in consequence of this cause.

(To be continued.)

## Figures and Tables

**Figure f1:**